# Cognitive Reserve and Executive Functions in Adults with Type 2 Diabetes

**DOI:** 10.1155/2020/7941543

**Published:** 2020-10-06

**Authors:** Paola Peña-González, Alejandra Mondragón-Maya, Juan Silva-Pereyra, Paloma Roa-Rojas

**Affiliations:** ^1^Dirección de Investigación, Instituto Nacional de Geriatría, Mexico; ^2^Carrera de Psicología, Facultad de Estudios Superiores Iztacala, Universidad Nacional Autónoma de México, Tlalnepantla, Estado de México, Mexico; ^3^Proyecto de Neurociencias, Facultad de Estudios Superiores Iztacala, Universidad Nacional Autónoma de México, Tlalnepantla, Estado de México, Mexico

## Abstract

**Background:**

Adults with type two diabetes mellitus (DM2) show cognitive deficits within the executive function domain. The detrimental effects of DM2 over executive function (EF) performance may be mediated by factors such as cognitive reserve (CR). CR mediates cognitive performance by delaying the appearance of clinical symptoms from subjacent brain pathology or attenuating the severity of such symptoms. Our main goal was to study the effects of CR on executive functions of adults with DM2.

**Methods:**

Data from a total of 1,034 adults were included (362 women, 672 men). Subjects were categorized into four groups: subjects with DM2 and high CR (*n* = 235), control subjects with high CR (*n* = 265), subjects with DM2 and low CR (*n* = 298), and control subjects with low CR (*n* = 236). CR was quantified through 3 proxies: education, occupational complexity, and leisure activities. Executive functions were evaluated through visual scanning, verbal fluency, and backwards counting tasks. First, a series of four one-way ANOVAs was performed where group was included as a between-subject factor and executive function as a dependent variable. Second, a hierarchical multiple regression analysis was conducted to assess the weight of each CR proxy on EF performance.

**Results:**

CR level significantly affected all executive function scores independently of the diabetes status. Hierarchical regression analyses indicated that years of education accounted for most of the variance in the model for executive function performance. In this study, we found that there is a significant effect of CR on executive function performance of DM2 subjects and education is the most important CR proxy.

## 1. Introduction

Type two diabetes mellitus (DM2) prevalence is growing worldwide [1]. In Mexico, the current prevalence of DM2 in older adults is 21.4% [2] and the prevalence among Mexican population 50 years and older is projected to increase up to 34% by 2050 [3].

Adults with DM2 show cognitive deficits within multiple domains [4, 5]. However, such impairment seems to be more consistent in the executive function domain [6]. For instance, in a recent meta-analysis, all aspects of executive function, including verbal fluency, mental flexibility, inhibition, working memory, and attention, were significantly associated with DM2 [7]. Nonetheless, substantial variability in executive function test performance has been found among adults with DM2. Selective impairment of executive control [8], phonemic fluency, cognitive flexibility, and processing speed [9] has been found. Also, some longitudinal studies have shown a poorer performance of adults with DM2 than healthy controls on executive functioning [10] and processing speed [11].

This type of evidence suggests that the detrimental effects of DM2 over executive function performance may be due to factors directly related with the disease, such as its duration or glycemic control [7]; however, performance can also be mediated by factors such as age [12] and cognitive reserve (CR). CR mediates cognitive performance by delaying the appearance of clinical symptoms from subjacent brain pathology or attenuating the severity of such symptoms [13, 14]. Previous findings have demonstrated the mediating effect of CR on executive functions [15, 16].

There is little evidence about the effect of CR on cognition of adults with diabetes. Evidence suggests that there is an association between educational level and cognitive performance in subjects with DM2. For instance, Guerrero-Berroa and cols. [17] found that higher education was associated with better cognitive performance in subjects with diabetes who were cognitively intact. Moreover, the CR effect has also been studied in health-related factors such as obesity [18–20]. Findings from these studies suggest that CR attenuates the cognitive deficits (i.e., attention, executive functions, and memory) related to obesity. Although evidence is not directly focused on diabetes, both conditions—DM2 and obesity—are two clinical entities strongly associated.

To study the effects of CR on executive functions of adults with DM2, we compared the performance of executive functions between adults without DM2 and high CR, adults without DM2 and low CR, adults with DM2 and high CR, and adults with DM2 and low CR. Thus, based on previous evidence, it would be expected that adults without DM2 will have a better performance in executive function tasks than adults with DM2. Moreover, those adults without DM2 and high CR will outperform the remaining three groups.

Additionally, since CR quantification relies on proxy measures [21] like years of formal education, occupational complexity, and leisure activities [22, 23], it is not clear yet which proxy has the greatest effect on executive function performance. Some studies [24] have addressed this issue by measuring the effect of some CR proxies like education and occupational complexity on cognition. These studies have reported that older adults with higher education had lower risk of dementia. Thus, we hypothesized that the effect of education would be bigger than the effect of occupational complexity on executive function performance of adults with DM2.

## 2. Materials and Methods

### 2.1. Subjects

Data were taken from 2012 Mexican Health and Ageing Study (MHAS). The MHAS is a prospective aging study that is aimed at assessing the impact of disease on health, functionality, and mortality of Mexican older adults. It was designed based on the framework of the Health and Retirement Study from the United States (HRS) [25]. The MHAS comprises multiple variables, including cognitive measures such as executive functions.

Data from a total of 1,034 Mexican adults were included in this study (362 women, 672 men) distributed into two groups: one group of adults with diabetes (*N* = 533) and a control group (*N* = 501) who at the time of the exploration did not have DM2. Inclusion criteria for the DM2 group included having self-reported diabetes and being over 56 years old. Individuals with cognitive impairment (having a standard total cognitive score below 70), any missing data about multimorbidity, depressive symptoms, or executive function measures were excluded from the analysis. Their age ranged from 56 to 93 years. To identify the presence of DM2, subjects answered if a physician or a medical professional had diagnosed them with diabetes. To assess multimorbidity, the presence of six diseases was considered (cancer, high blood pressure, lung disease, heart problems, stroke, and arthritis). This information was collected during direct interviews with the participants or their caregiver. Control group subjects were selected randomly based on inclusion criteria and characteristics of adults with DM2.

The group of subjects with diabetes and the control group were divided into two more groups according to their CR scores. Data of subjects with a CR composite above 100 were considered having “high reserve,” and those with a total score below 100 were considered having “low reserve.” Four groups were obtained: subjects with DM2 and high CR (*n* = 235), control subjects with high CR (*n* = 265), subjects with DM2 and low CR (*n* = 298), and control subjects with low CR (*n* = 236). The sociodemographic variables included in the study were as follows: age (56 years old); gender (female versus male); and marital status (married, partnered, separated, divorced, widowed, and never married).

### 2.2. Measures

#### 2.2.1. Cognitive Reserve

CR was quantified through a recently validated questionnaire called CORQ-MEX (Cognitive Reserve Questionnaire-Mexico) (P Roa-Rojas 2017, unpublished data). CORQ-MEX quantifies CR through 3 proxies: education (EDU), occupational complexity (OCOM), and leisure activities (LECA). The Cronbach alpha results showed an internal consistency coefficient of 0.70 for all LECA items. Cronbach's alpha for EDU, OCOM, LECA, and TOT CORQ-MEX was 0.78. Education was measured by 1 item; subjects reported the total number of years at school and university attendance. Occupational complexity was measured by 1 item; participants indicated their main occupation during their last recent years, which was computed into a 1 to 5 gradient depending on its complexity, 1 being the least complex and 5 being the most complex [26]. Leisure activities were measured with 13 items, which included daily activities (4 items), incidental physical activities (5 items), social activities (2 items), and parental educational level (2 items). For each item, participants answered the frequency of engagement into these activities and then, each item was transformed into a dichotomous response (i.e., no = 0; yes = 1).

Raw scores from each proxy were transformed into standard scores (ST). First, they were transformed into *Z* values and then, the obtained values were rescaled to a mean of 100 and a standard deviation of 15. Also, a total score or composite was obtained by calculating the average of the three proxies' ST.

#### 2.2.2. Executive Functions

To assess the executive function domain, visual scanning, verbal fluency, and backwards counting tasks were included. These tasks were previously validated and normalized for the Mexican population [27]. The visual scanning task required subjects to detect stimuli (up to 60) embedded among other similar stimuli as quickly as possible in a maximum period of 60 seconds. The total score was the number of visual stimuli correctly selected. Verbal fluency was measured using a semantic fluency task. The task required subjects to name animals for one minute, without word repeating. The total score was the number of animal names correctly said. The backwards counting task required subjects to count backwards from 20 to 0 as quickly as possible in a 60-second period. The maximum score was two points, which were obtained if the task was performed successfully on the first try; one point was obtained if the participant performed successfully on the second try, and no points were given if the subject failed on either try. Finally, to assess the executive function domain as a whole, a composite was created with the sum of the three tasks' scores.

#### 2.2.3. Depressive Symptoms

Considering that adults with DM2 are at high risk of developing mood disorders [28], we evaluated the presence of clinically significant depressive symptoms with the nine-item version of the depression scale of the Center for Epidemiologic Studies (CES-D). This version has been validated in a Mexican elderly population [29]. This instrument includes questions that assess dysphoric mood, motivation, concentration, loss of pleasure, and poor sleep.

### 2.3. Procedure

Data from MHAS was collected through structured interviews performed at the participant's address. The interview comprised several sections, which aimed to assess demographic, health, cognitive, and functional variables, among others. The cognitive section included executive function tests, which were administered in the following order: visual scanning, verbal fluency, and backwards counting. The interview was performed by trained health-related professionals and lasted three hours approximately. Detailed information about MHAS is described elsewhere (https://www.enasem.org/).

### 2.4. Statistical Analyses

Data were analyzed using SPSS v.20. To address the first aim of the study, a series of four one-way ANOVAs was performed where group was included as a between-subject factor (four groups) and executive function (one analysis per executive function tasks and the composite score) as a dependent variable. Then, *post hoc* tests (Tukey's Honestly Significant Difference method) were used for pairwise comparisons.

For the second purpose, a hierarchical multiple regression analysis was conducted to assess the weight of each CR proxy on executive function performance. Taking executive function composite as a dependent variable, the demographic, clinical, and CR proxy variables were entered sequentially into the regression model. Demographic variables were entered first, clinical variables were added in the second step, and education, occupational complexity, and leisure activities were added in the third and final step.

## 3. Results

Table 1 presents a summary of the sociodemographic and clinical characteristics of the whole sample. No significant differences between adults with DM2 (mean age 65) and control subjects (mean age 64) were observed in age (0.121), gender (0.54), and marital status (0.93). Regarding multimorbidity, there were no significant differences in cancer (0.60), lung disease (0.39), and arthritis (0.08) prevalence between the two groups; however, there were significant differences in high blood pressure (0.000), heart problems (.002), and stroke (0.000). 64% of adults with diabetes had high blood pressure, 8.2% heart problems, and 5.2% stroke while in the control group, 40% had high blood pressure, 3.5% heart problems, and 0.99% stroke. Also, there were significant differences in depressive symptom (.003) prevalence between the two groups.

Table 2 shows the mean scores and standard deviations of each group for cognitive reserve proxies (EDU, OCOM, and LECA), the executive function tasks, and the composite measure. The CR level significantly affected all executive function scores independently of the diabetes status. In other words, subjects with and without DM2 and high CR level outperformed subjects with a low CR level with or without diabetes in visual scanning (*F*(3, 1030) = 55.3, *p* < 0.0001), verbal fluency (*F*(3, 1030) = 33.9, *p* < 0.0001), backwards counting (*F*(3, 1030) = 11.9, *p* < 0.0001), and the executive function composite score (*F*(3, 1030) = 63, *p* < 0.0001). *Post hoc* analyses revealed significant differences between subjects with and without DM2 only if they had low CR. *Post hoc* results are shown in Table 3.

EDU ST: standard education; OCOM ST: standard occupational complexity; LECA ST: standard leisure activities; GCON: control group; GDIAB: diabetic group.

The hierarchical regression analysis indicated that when CR proxy variables were added to the model in the final step, a greater amount of variance was explained; however, not all predictors were independently significant; to see these results, go to Table 4. With regard to demographic variables, in the first step, age, gender, and marital status together explained 8% of the variance, with age, gender, and marital status as significant predictors of executive function performance in the first step. The addition of clinical variables (CES-D score and multimorbidity) resulted in a significant *F* change and increased the variance explained up to 10%, with age and multimorbidity as significant predictors of executive function performance in the second step. The full model accounted for 34% of the variance in executive function performance (*F* (8,521) = 36.2, *p* < 0.0001) with age, education, leisure activities, and occupational complexity as significant predictors in the final step. This indicates that greater scores in CR proxies are associated with better performance in executive function tasks. However, it is probable that most of this variance was accounted for by education (beta = 0.31, *t* (8,521) = 6.6, *p* < 0.0001), the most significant predictor of executive functions in the full model.

## 4. Discussion

The first aim of the present study was to compare the executive function performance of participants with and without DM2 regarding their CR level. According to the results of the first analyses, we partially accept the hypothesis. We found differences between high-CR and low-CR participants irrespectively of their diabetes status. We expected to observe significant differences between the DM2 groups and controls, since evidence has shown the detrimental effect of DM2 on neurocognition [30]. However, we found no differences between DM2 and control performance unless CR was considered. Thus, our results show that CR seems to be a stronger modulating factor than the diabetes status regarding executive function performance. Although evidence has pointed out that DM2 is related to a decrement in executive function performance [7, 8], the effect sizes of such changes tend to be small in adults under 60 years old and become moderate in people over 65 [31]. Biessels et al. [32] suggested that the clinical cognitive impairment associated with diabetes may arise at some critical periods in lifetime: during the first 7 years of life, when normal neurodevelopment is occurring, and after 65 years old, when neurodegenerative changes associated with aging are taking place. These authors explain that subjects with diabetes who did not develop the condition during such critical periods tend to show subtle cognitive changes, which may not reach clinical relevance. Since people aged 56 and older comprised our sample, the cognitive changes associated with DM2 could have been kept at a subtle level and/or the heterogeneity of the performance among the individuals could blur the diabetes status effect. Moreover, some studies have indicated that marked cognitive decrements associated with DM2 tend to be influenced by factors directly related with the disease like early DM2 onset, poor glycemic control, severe hypoglycemic episodes during the early years, and presence of micro- and macrovascular diseases [32–34]. In the present study, the sample's missing values regarding such information prevented us from performing further analyses.

As mentioned above, little is known about the effect of CR on executive functions of subjects with diabetes. Our results are consistent with Guerrero-Berroa et al. [17] regarding the mediating effect of education—a CR proxy—on cognition of subjects with diabetes. Such effect was observed in all cognitive domains, including executive functions. Our results are also consistent with the studies that explored the association between CR and cognition in obesity, a condition strongly related to DM2 [18, 20].

Regarding the second aim of the study, hierarchical regression analyses indicated that years of education accounted for most of the variance in the model for executive function performance. Such findings support our second hypothesis, since we predicted that the effect of education on executive function performance would be greater than the effect of the other CR proxies we assessed (i.e., occupational complexity and leisure activities). These results are consistent with those reported by Ihle et al. [20] in subjects with obesity. They found that the cognitive impairment associated with obesity was reduced when years of education in early life was taken into account. Moreover, a study performed by Jefferson et al. [35], which aimed to determine the contribution of each CR proxy measure (i.e., education, socioeconomic status, reading ability, and cognitive activities) on late-life cognitive functioning of healthy participants, indicated that education and reading abilities were the strongest CR proxies related to cognition in healthy elderly. Our findings suggest that education may play a crucial role for executive function performance in later life, which supports the CR hypothesis. Education in early life may stimulate and enhance the functional efficiency of the cognitive system. Such functional changes may be perdurable through life, becoming evident at old age and in the presence of pathology [36]. Indeed, there is plenty of evidence supporting the causal relationship between educational attainment and cognition in adult ages [37–39]. Additionally, some studies have explored the relationship between educational attainment and treatment adherence in different pathologies. A recent systematic review on this topic indicated that higher education along with employment has a positive effect on adherence [40]. We consider that such findings are relevant for our study, since it is possible that high-educated subjects with DM2 may have been more engaged with their medical treatment, thus decreasing the detrimental DM2 effects on neurocognition.

## 5. Study Limitations

We have limited information about factors directly related with the disease like DM2 onset or glycemic control. Future studies must address this issue and consider factors directly related with DM2 such as glycemic control to better understand the mechanisms through which DM2 has a detrimental effect on cognitive functions such as executive functions and the processes by which CR moderates these effects. Moreover, considering our study is cross-sectional, there is a need for longitudinal studies that may provide a better understanding of the effect of CR on executive functions of adults with DM2.

## 6. Conclusions

In this study, we found that there is a significant effect of CR on executive functions of subjects with DM2. Education is the most important CR proxy. We suggest that the effect of education on cognition of subjects with DM2 may be due to (1) the enhanced neural efficiency related to educational attainment during early years and (2) a better adherence to treatment in those subjects with more years of education, which may have reduced the cognitive detrimental effect associated to DM2.

## Figures and Tables

**Table 1:** Sociodemographic and clinical characteristics of the study sample (*N* = 1033).

		Total (*N* = 1,034)	Nondiabetic (*N* = 501)	Diabetic (*N* = 533)
		*N*	*N*	*N*
Age	56-61	383	194	189
	62-65	274	116	158
	66-71	223	111	112
	72-92	154	80	74

Gender	Male	672	325	347
	Female	362	176	186

Marital status	Married	684	317	367
	Partnered	80	44	36
	Separated	58	27	31
	Divorced	22	18	4
	Widowed	155	72	83
	Never married	35	23	12

Multimorbidity	Cancer	34	15	19
	High blood pressure	545	201	344
	Lung disease	99	44	55
	Heart problems	62	18	44
	Stroke	33	5	28
	Arthritis	235	102	133

CESD-9	Mean total score	3.1	2.9	3.4

Cognitive reserve	High CR	500	265	235
	Low CR	534	236	298

**Table 2:** Group comparisons on cognitive reserve proxies and executive function performance.

	High reserve		Low reserve		Total (*N* = 1033)
	GCON (*N* = 265)	GDIAB (*N* = 235)	GCON (*N* = 236)	GDIAB (*N* = 298)	
EDU ST	115 (16.9)	114 (16.4)	92.2 (8.2)	92.2 (9.2)	103 (17.3)
OCOM ST	111 (10.9)	111 (11.5)	97.2 (12.8)	92 (13.9)	99 (15.2)
LECA ST	107 (16.2)	108 (18)	92.8 (7.8)	92 (7.7)	102 (15.1)
Working memory	1.9 (0.38)	1.9 (0.36)	1.7 (0.69)	1.7 (0.64)	1.8 (0.55)
Verbal fluency	17.5 (5.13)	16.6 (5.2)	13.8 (4.8)	14.3 (4.6)	15.5 (5.1)
Attention	35.8 (15.3)	33.8 (14.5)	24.2 (14.4)	23.2 (12.3)	29.1 (15.1)
Executive composite	55.34 (18.3)	52.3 (17.4)	39.8 (17.5)	39.2 (15.04)	46.4 (18.5)

**Table 3:** Post hoc comparisons using Tukey's HSD. Mean differences shown.

		GCON HIGH RES	GDIAB HIGH RES	GCON LOW RES	GDIAB LOW RES
GCON HIGH RES (*N* = 265)	Verbal fluency	1	0.88	3.7^∗^	3.2^∗^
	Attention		2	11.6^∗^	12.6^∗^
	Working memory		0.003	0.21^∗^	0.201^∗^
	Executive comp		2.9	15.5^∗^	16^∗^

GDIAB HIGH RES (*N* = 235)	Verbal fluency		1	2.8^∗^	2.3^∗^
	Attention			9.5^∗^	10.5^∗^
	Working memory			0.207^∗^	0.198^∗^
	Executive comp			12.5^∗^	13.13^∗^

GCON LOW RES (*N* = 236)	Verbal fluency			1	0.442
	Attention				1.01
	Working memory				0.009
	Executive comp				0.56

GDIAB low RES (*N* = 298)	Verbal fluency				1
	Attention				
	Working memory				
	Executive comp				

GCON HIGH RES: control group with high reserve; GDIAB HIGH RES: diabetic group with high reserve; GCON LOW RES: control group with low reserve; GDIAB LOW RES: diabetic group with low reserve. ^∗^The mean difference is significant at the 0.05 level.

**Table 4:** Multiple regressions predicting executive function performance.

Predictors	Verbal fluency *R*^2^ = 0.15		Attention *R*^2^ = 0.32		Working memory *R*^2^ = 0.07		Executive comp *R*^2^ = 0.34	
	*B*	*T*	*B*	*T*	*B*	*T*	*B*	*T*
Age	-0.16	-3.6∗	-0.28	-7^∗^	-0.12	-2.6^∗^	-0.28	-7.1^∗^
Gender	-0.05	0.1.2	0.03	0.75	-0.13	-2.7^∗^	0.00	0.12
Marital status	-0.04	-1.09	-0.04	-1.1	-0.001	-0.02	-0.05	-1.3
Multimorbidity	0.02	0.43	0.01	0.29	-0.08	-1.7	0.01	0.32
CESD-9	0.06	1.4	-0.04	-1.1	0.01	0.32	-0.01	-0.44
Education	0.31	6.6^∗^	0.38	9.2^∗^	0.16	3.4^∗^	0.41	10^∗^
Leisure act	0.11	2.4^∗^	0.09	2.3^∗^	-0.03	-0.77	0.11	2.7^∗^
Occupational complexity	0.05	1.1	0.13	3.3^∗^	0.06	1.3	0.12	3.2^∗^

^∗^The mean difference is significant at the 0.05 level.

## Data Availability

The quantitative data supporting this study comes from the MHAS (Mexican Health and Aging Study) dataset. Data files and documentation are for public use and available at https://www.MHASweb.org. This has been cited in Materials and Methods of the study. The processed data are available from the corresponding author upon request.
